# Copy of a Letter from Mr. Weston, Surgeon, of St. Ann's Bay, Jamaica, to Mr. Ring, Dated Sept. 19, 1803

**Published:** 1803-12-01

**Authors:** 


*Copy of a Letter from Mr. Weston, Surgeon, of St. Ann's Bay, Jamaica, to Mr. Ring, dated Sept. 19, 1803.*

SIR,

EMBOLDENED by my experience of your goodness, I take the liberty to request you will be pleased to send me, per packet, (which sails monthly) a few ivory points coated with vaccine matter.

After I had the honour of being introduced to you in London, by my friend Mr. Wilson, you sedulously and most benevolently instructed me in every thing that appertained to the vaccine disease. A spark of your philanthropic ardour fired my mind; and I came hither with sanguine expectations of diffusing and perpetuating (what I conceive) the greatest good which God in his benevolence has given to mankind.

With

With the matter I obtained from you on ivory points, I inoculated my own children, and twenty others, the day after my arrival. I roused the public attention to vaccination by my communications in the periodical works of this country; and propagated the matter to every part of the island. I received by letter from upwards of sixty practitioners, congratulations on the re-appearance of the vaccine disease. But, alas! the hot season set in; and I was mortified and disappointed in being obliged to acknowledge the lamentable fact, that in a temperature of 90, the vaccine matter loses its activity, and becomes absolutely effete. Assured *now* of this fact, I have presumed to ask a supply of you. We have eight months of the year, in which the thermometer is below 80; I therefore propose to propagate the disease on the coast during the winter; and in the summer months translate it to the mountains, where the temperature is occasionally under 70. I am prompted to this exertion by a consciousness, that with pains we may perpetuate the vaccine matter in this country; and I have ascertained beyond cavil, that the Negro habit is susceptible of the disease; having in several hundred instances seen it in them characteristically marked. My knowledge of the great sacrifices you have made to disseminate this benign practice, is a sufficient assurance that you will pardon this trouble.

